# *trans*-Bis[4-amino-*N*-(pyrimidin-2-yl-κ*N*)benzene­sulfonamidato-κ*N*]bis(*N*,*N*-dimethyl­formamide-κ*O*)cobalt(II)

**DOI:** 10.1107/S160053681204336X

**Published:** 2012-10-24

**Authors:** Jing Jing Guo, Wei Wang, Yi Dong Zhang, Li Yang, Shu Hua Zhang

**Affiliations:** a College of Chemistry and Bioengineering, Guilin University of Technology, Guilin 541004, People’s Republic of China

## Abstract

The title complex, [Co(C_10_H_9_N_4_O_2_S)_2_(C_3_H_7_NO)_2_], lies across an inversion center. The Co^II^ atom is coordinated in a slightly distorted octa­hedral geometry by four N atoms from two bidentate 4-amino-*N*-(pyrimidin-2-yl)benzene­sulfonamidate (sulfadiazine) anions and two O atoms from two dimethyl­formamide (DMF) ligands. The dihedral angle between the benzene and pyrimidine rings is 82.37 (13)°. A three-dimensional network is generated by N—H⋯O hydrogen bonds between the amino groups and of the sulfonamidate O atoms of neighbouring mol­ecules. The DMF ligand is disordered over two sets of sites in a 0.559 (4):0.441 (4) ratio.

## Related literature

For background to sulfonamides, see: Connor (1998 ▶). For background to metal complexes of sulfadiazine, see: Wang *et al.* (2009 ▶, 2010 ▶); Ajibade *et al.* (2006 ▶); Hossain *et al.* (2011 ▶); Tommasino *et al.* (2011 ▶); Ghosh *et al.* (2011 ▶).
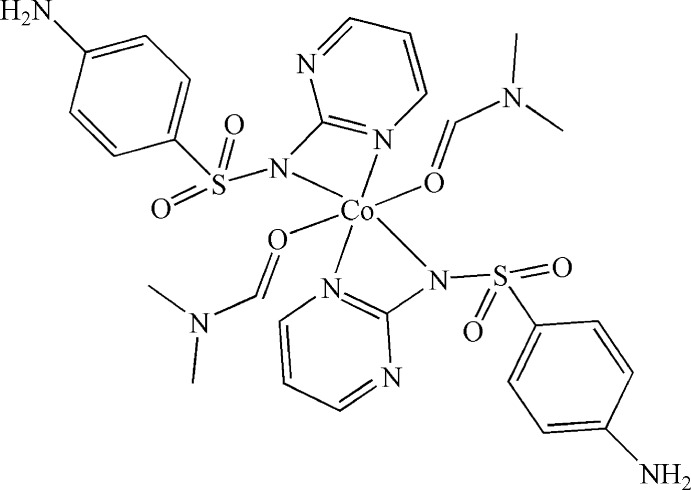


## Experimental

### 

#### Crystal data

[Co(C_10_H_9_N_4_O_2_S)_2_(C_3_H_7_NO)_2_]
*M* *_r_* = 703.67
Monoclinic, 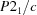

*a* = 8.9008 (6) Å
*b* = 11.2078 (6) Å
*c* = 16.5565 (9) Å
β = 102.147 (6)°
*V* = 1614.67 (16) Å^3^
*Z* = 2
Mo *K*α radiation
μ = 0.72 mm^−1^
*T* = 298 K
0.28 × 0.25 × 0.20 mm

#### Data collection

Bruker SMART CCD diffractometer
Absorption correction: multi-scan (*SADABS*; Bruker, 2001 ▶) *T*_min_ = 0.815, *T*_max_ = 0.869
13220 measured reflections
3495 independent reflections
2989 reflections with *I* > 2σ(*I*)
*R*_int_ = 0.028

#### Refinement

*R*[*F* ^2^ > 2σ(*F* ^2^)] = 0.040
*wR*(*F* ^2^) = 0.095
*S* = 0.99
3495 reflections
222 parameters
36 restraints
H-atom parameters constrained
Δρ_max_ = 0.33 e Å^−3^
Δρ_min_ = −0.35 e Å^−3^

### 

Data collection: *SMART* (Bruker, 2001 ▶); cell refinement: *SAINT* (Bruker, 2001 ▶); data reduction: *SAINT*; program(s) used to solve structure: *SHELXS97* (Sheldrick, 2008 ▶); program(s) used to refine structure: *SHELXL97* (Sheldrick, 2008 ▶); molecular graphics: *SHELXTL* (Sheldrick, 2008 ▶); software used to prepare material for publication: *SHELXTL*.

## Supplementary Material

Crystal structure: contains datablock(s) I, global. DOI: 10.1107/S160053681204336X/wm2693sup1.cif

Structure factors: contains datablock(s) I. DOI: 10.1107/S160053681204336X/wm2693Isup2.hkl

Additional supplementary materials:  crystallographic information; 3D view; checkCIF report

## Figures and Tables

**Table 1:** Selected bond lengths (Å)

Co1—O1	2.065 (19)
Co1—N1	2.121 (2)
Co1—N2	2.1460 (18)

**Table 2:** Hydrogen-bond geometry (Å, °)

*D*—H⋯*A*	*D*—H	H⋯*A*	*D*⋯*A*	*D*—H⋯*A*
N4—H4*B*⋯O3^i^	0.86	2.31	3.112 (3)	155
N4—H4*C*⋯O3^ii^	0.86	2.27	2.951 (3)	136

Symmetry codes: (i) 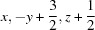; (ii) 
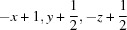
.

**Fig. 1.**
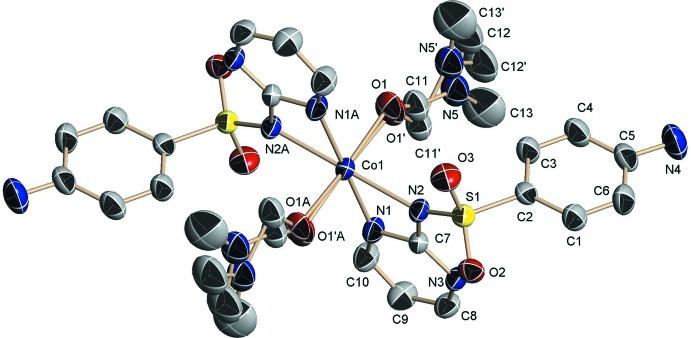
The molecular structure of (I), showing 30% probability displacement ellipsoids. [Symmetry code (a); -x, 1 - y, -z.] Both parts of the disordered DMF ligand are shown.

**Fig. 2.**
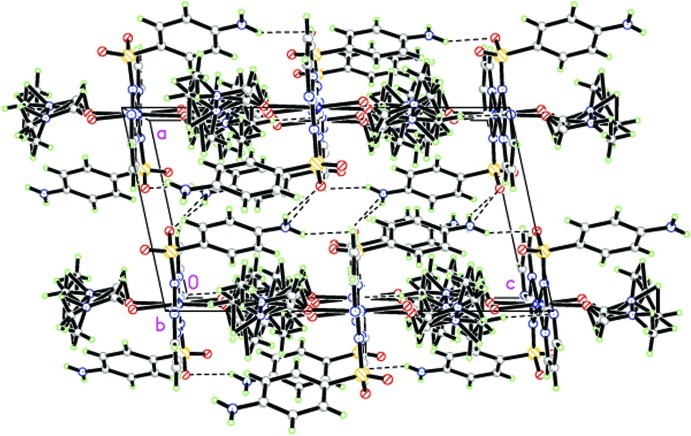
Packing diagram of (I) viewed down [010]. Dashed lines denote N—H···O hydrogen bonds.

## supplementary crystallographic information

### Comment

Sulfonamides are among the most widely used antibacterial agents (Connor, 1998), because of their low cost, low toxicity, and excellent activity against bacterial diseases. Sulfadiazine, or 4-amino-*N*-pyrimidin-2-yl-benzenesulfonamide, is a sulfanilamide antibiotic and its metal complexes have been studied previously (Wang *et al.* 2009, 2010; Ajibade *et al.*, 2006; Hossain *et al.*, 2011; Tommasino *et al.*, 2011; Ghosh *et al.*, 2011). The crystal structure of the title compound, [Co(C_10_H_9_N_4_O_2_S)_2_(C_3_H_7_NO)_2_], a cobalt sulfadiazine complex with additional dimethylformamide ligands, (I), is presented herein.

The molecular structure of (I) is shown in Fig. 1. The Co^II^ ion lies on a centre of inversion and is coordinated by four N atoms from two symmetry-related 4-amino-*N*-pyrimidin-2-yl-benzenesulfonamidate anions and two O atoms from two dimethylformamide ligands, forming a slightly distorted octahedral N_4_O_2_ geometry (Table 1). The dihedral angle between the phenyl and pyrimidine rings is 82.37 (13) °. A three-dimensional hydrogen-bonded network is generated by N—H···O interactions (Table 2; Fig.2).

### Experimental

Complex (I) was prepared from a mixture of sulfadiazine (1 mmol, 0.250 g), Co(NO_3_)_2_^.^6H_2_O (0.5 mmol, 0.145 g), triethylamine (0.5 ml) and *N*,*N*-dimethylformamid (8 ml) sealed in a 15 ml teflon-lined stainless steel bomb, and kept at 373 K for 96 h under autogenous pressure. After the reaction was slowly cooled to room temperature, red block-like crystals were obtained (yield: 72% based on cobalt). Anal./calc. for C_26_H_32_N_10_CoO_6_S_2_(%): C 44.38; H 4.58; N 19.90. Found(%): C 44.34; H 4.61; N 19.95.

### Refinement

H atoms were positioned geometrically and refined with a riding model, with distances 0.86 Å (N—H), 0.96 Å (CH_3_) or 0.93 Å (aromatic ring), and with *U*_iso_(H) = 1.2 *U*_eq_(aromatic ring, N—H) or *U*_iso_(H) = 1.5 *U*_eq_(CH_3_). The DMF ligand is positionally disordered over two sets of sites in a 0.559 (4):0.441 (4) ratio.

### Crystal data

**Table:**

[Co(C_10_H_9_N_4_O_2_S)_2_(C_3_H_7_NO)_2_]	*F*(000) = 730
*M**_r_* = 703.67	*D*_x_ = 1.447 Mg m^−^^3^
Monoclinic, *P*2_1_/*c*	Mo *K*α radiation, λ = 0.71073 Å
Hall symbol: -P 2ybc	Cell parameters from 4388 reflections
*a* = 8.9008 (6) Å	θ = 3.0–28.7°
*b* = 11.2078 (6) Å	µ = 0.72 mm^−^^1^
*c* = 16.5565 (9) Å	*T* = 298 K
β = 102.147 (6)°	Block, red
*V* = 1614.67 (16) Å^3^	0.28 × 0.25 × 0.20 mm
*Z* = 2	

### Data collection

**Table:**

Bruker SMART CCD diffractometer	3495 independent reflections
Radiation source: fine-focus sealed tube	2989 reflections with *I* > 2σ(*I*)
Graphite monochromator	*R*_int_ = 0.028
Detector resolution: 0 pixels mm^-1^	θ_max_ = 27.0°, θ_min_ = 3.0°
phi and ω scans	*h* = −11→11
Absorption correction: multi-scan (*SADABS*; Bruker, 2001)	*k* = −14→13
*T*_min_ = 0.815, *T*_max_ = 0.869	*l* = −21→19
13220 measured reflections	

### Refinement

**Table:**

Refinement on *F*^2^	Secondary atom site location: difference Fourier map
Least-squares matrix: full	Hydrogen site location: inferred from neighbouring sites
*R*[*F*^2^ > 2σ(*F*^2^)] = 0.040	H-atom parameters constrained
*wR*(*F*^2^) = 0.095	*w* = 1/[σ^2^(*F*_o_^2^) + (0.0311*P*)^2^ + 1.5416*P*] where *P* = (*F*_o_^2^ + 2*F*_c_^2^)/3
*S* = 0.99	(Δ/σ)_max_ < 0.001
3495 reflections	Δρ_max_ = 0.33 e Å^−^^3^
222 parameters	Δρ_min_ = −0.35 e Å^−^^3^
36 restraints	Extinction correction: *SHELXL97* (Sheldrick, 2008), Fc^*^=kFc[1+0.001xFc^2^λ^3^/sin(2θ)]^-1/4^
Primary atom site location: structure-invariant direct methods	Extinction coefficient: 0.0012 (1)

### Special details

**Table:**

Geometry. All e.s.d.'s (except the e.s.d. in the dihedral angle between two l.s. planes) are estimated using the full covariance matrix. The cell e.s.d.'s are taken into account individually in the estimation of e.s.d.'s in distances, angles and torsion angles; correlations between e.s.d.'s in cell parameters are only used when they are defined by crystal symmetry. An approximate (isotropic) treatment of cell e.s.d.'s is used for estimating e.s.d.'s involving l.s. planes.
Refinement. Refinement of *F*^2^ against ALL reflections. The weighted *R*-factor *wR* and goodness of fit *S* are based on *F*^2^, conventional *R*-factors *R* are based on *F*, with *F* set to zero for negative *F*^2^. The threshold expression of *F*^2^ > σ(*F*^2^) is used only for calculating *R*-factors(gt) *etc*. and is not relevant to the choice of reflections for refinement. *R*-factors based on *F*^2^ are statistically about twice as large as those based on *F*, and *R*- factors based on ALL data will be even larger.

### Fractional atomic coordinates and isotropic or equivalent isotropic displacement parameters (Å^2^)

**Table:**

	*x*	*y*	*z*	*U*_iso_*/*U*_eq_	Occ. (<1)
C1	0.4095 (3)	0.9002 (2)	0.13724 (16)	0.0476 (6)	
H1A	0.4447	0.9344	0.0935	0.057*	
C2	0.3251 (2)	0.7957 (2)	0.12498 (14)	0.0363 (5)	
C3	0.2761 (3)	0.7454 (3)	0.19139 (15)	0.0507 (6)	
H3A	0.2212	0.6742	0.1843	0.061*	
C4	0.3079 (3)	0.7994 (3)	0.26800 (16)	0.0572 (7)	
H4A	0.2744	0.7640	0.3119	0.069*	
C5	0.3892 (3)	0.9061 (3)	0.28047 (16)	0.0508 (7)	
C6	0.4421 (3)	0.9543 (3)	0.21390 (17)	0.0568 (7)	
H6A	0.5002	1.0240	0.2213	0.068*	
C7	−0.0153 (2)	0.73277 (19)	0.00525 (13)	0.0319 (5)	
C8	−0.1683 (3)	0.8945 (2)	−0.00174 (16)	0.0438 (6)	
H8A	−0.1808	0.9767	0.0005	0.053*	
C9	−0.2982 (3)	0.8242 (2)	−0.01745 (18)	0.0513 (7)	
H9A	−0.3961	0.8573	−0.0260	0.062*	
C10	−0.2766 (3)	0.7031 (2)	−0.01992 (17)	0.0497 (6)	
H10A	−0.3616	0.6528	−0.0293	0.060*	
Co1	0.0000	0.5000	0.0000	0.03466 (14)	
N1	−0.1368 (2)	0.65637 (17)	−0.00916 (13)	0.0402 (5)	
N2	0.1174 (2)	0.66834 (16)	0.01562 (12)	0.0350 (4)	
N3	−0.0252 (2)	0.85105 (17)	0.01044 (12)	0.0372 (4)	
N4	0.4124 (3)	0.9634 (3)	0.35511 (15)	0.0724 (8)	
H4B	0.3765	0.9335	0.3950	0.087*	
H4C	0.4629	1.0294	0.3621	0.087*	
O2	0.29575 (19)	0.81638 (15)	−0.03312 (10)	0.0437 (4)	
O3	0.38724 (19)	0.62601 (16)	0.02969 (12)	0.0519 (5)	
S1	0.28414 (6)	0.72636 (5)	0.02742 (3)	0.03470 (15)	
O1	0.0274 (19)	0.4809 (16)	0.1263 (12)	0.053 (2)	0.559 (4)
C11	−0.0332 (9)	0.5365 (7)	0.1739 (6)	0.0563 (17)	0.559 (4)
H11A	−0.1225	0.5630	0.1388	0.084*	0.559 (4)
C13	−0.1386 (10)	0.6711 (8)	0.2810 (5)	0.107 (2)	0.559 (4)
H13A	−0.1134	0.6764	0.3402	0.160*	0.559 (4)
H13B	−0.1218	0.7471	0.2577	0.160*	0.559 (4)
H13C	−0.2446	0.6489	0.2632	0.160*	0.559 (4)
C12	0.0603 (12)	0.5055 (8)	0.3034 (5)	0.098 (2)	0.559 (4)
H12A	0.1286	0.4397	0.3204	0.147*	0.559 (4)
H12B	0.1008	0.5758	0.3334	0.147*	0.559 (4)
H12C	−0.0388	0.4876	0.3145	0.147*	0.559 (4)
N5	−0.0499 (7)	0.5829 (5)	0.2463 (3)	0.0665 (13)	0.559 (4)
O1'	−0.009 (3)	0.501 (2)	0.1318 (16)	0.053 (2)	0.441 (4)
C11'	−0.0812 (12)	0.5748 (10)	0.1647 (7)	0.0563 (17)	0.441 (4)
H11B	−0.1748	0.6128	0.1375	0.084*	0.441 (4)
C13'	0.1448 (14)	0.4546 (11)	0.2886 (6)	0.107 (2)	0.441 (4)
H13D	0.1783	0.4035	0.2493	0.160*	0.441 (4)
H13E	0.2274	0.5065	0.3134	0.160*	0.441 (4)
H13F	0.1146	0.4068	0.3306	0.160*	0.441 (4)
C12'	−0.0588 (13)	0.5888 (10)	0.3139 (5)	0.098 (2)	0.441 (4)
H12D	−0.0379	0.5933	0.3731	0.147*	0.441 (4)
H12E	−0.0382	0.6648	0.2916	0.147*	0.441 (4)
H12F	−0.1647	0.5681	0.2937	0.147*	0.441 (4)
N5'	0.0174 (10)	0.5244 (7)	0.2573 (5)	0.0665 (13)	0.441 (4)

### Atomic displacement parameters (Å^2^)

**Table:**

	*U*^11^	*U*^22^	*U*^33^	*U*^12^	*U*^13^	*U*^23^
C1	0.0493 (14)	0.0501 (15)	0.0422 (13)	−0.0135 (12)	0.0066 (11)	0.0020 (12)
C2	0.0331 (11)	0.0383 (12)	0.0342 (11)	−0.0024 (9)	−0.0004 (9)	0.0011 (10)
C3	0.0565 (15)	0.0515 (16)	0.0409 (13)	−0.0211 (13)	0.0031 (11)	0.0010 (12)
C4	0.0653 (17)	0.0698 (19)	0.0340 (13)	−0.0220 (15)	0.0047 (12)	0.0016 (13)
C5	0.0473 (14)	0.0605 (17)	0.0386 (13)	−0.0069 (12)	−0.0049 (11)	−0.0041 (12)
C6	0.0605 (17)	0.0523 (16)	0.0527 (16)	−0.0222 (14)	0.0006 (13)	−0.0068 (13)
C7	0.0351 (11)	0.0280 (11)	0.0313 (11)	−0.0031 (9)	0.0038 (9)	−0.0032 (9)
C8	0.0448 (13)	0.0333 (12)	0.0533 (15)	0.0049 (10)	0.0103 (11)	−0.0007 (11)
C9	0.0366 (12)	0.0485 (15)	0.0697 (18)	0.0047 (11)	0.0130 (12)	0.0027 (13)
C10	0.0359 (13)	0.0469 (15)	0.0657 (17)	−0.0097 (11)	0.0095 (12)	−0.0025 (13)
Co1	0.0448 (3)	0.0246 (2)	0.0328 (2)	−0.00372 (17)	0.00405 (18)	−0.00398 (17)
N1	0.0384 (10)	0.0322 (10)	0.0489 (12)	−0.0077 (8)	0.0066 (9)	−0.0043 (9)
N2	0.0353 (9)	0.0254 (9)	0.0416 (10)	−0.0006 (7)	0.0021 (8)	−0.0041 (8)
N3	0.0375 (10)	0.0269 (9)	0.0457 (11)	−0.0010 (8)	0.0056 (8)	−0.0031 (8)
N4	0.0836 (18)	0.0854 (19)	0.0428 (13)	−0.0255 (16)	0.0012 (13)	−0.0148 (13)
O2	0.0474 (9)	0.0473 (10)	0.0369 (9)	−0.0027 (8)	0.0099 (7)	0.0049 (8)
O3	0.0441 (9)	0.0492 (11)	0.0617 (11)	0.0162 (8)	0.0092 (8)	−0.0004 (9)
S1	0.0324 (3)	0.0339 (3)	0.0365 (3)	0.0030 (2)	0.0044 (2)	0.0001 (2)
O1	0.078 (7)	0.052 (5)	0.033 (2)	−0.005 (4)	0.020 (4)	−0.008 (3)
C11	0.062 (4)	0.067 (4)	0.041 (2)	−0.019 (3)	0.014 (3)	−0.008 (3)
C13	0.140 (5)	0.126 (5)	0.068 (3)	0.001 (4)	0.051 (3)	0.000 (3)
C12	0.128 (5)	0.118 (5)	0.049 (3)	−0.017 (4)	0.020 (3)	−0.012 (3)
N5	0.089 (3)	0.070 (3)	0.0436 (18)	−0.019 (2)	0.021 (2)	−0.007 (2)
O1'	0.078 (7)	0.052 (5)	0.033 (2)	−0.005 (4)	0.020 (4)	−0.008 (3)
C11'	0.062 (4)	0.067 (4)	0.041 (2)	−0.019 (3)	0.014 (3)	−0.008 (3)
C13'	0.140 (5)	0.126 (5)	0.068 (3)	0.001 (4)	0.051 (3)	0.000 (3)
C12'	0.128 (5)	0.118 (5)	0.049 (3)	−0.017 (4)	0.020 (3)	−0.012 (3)
N5'	0.089 (3)	0.070 (3)	0.0436 (18)	−0.019 (2)	0.021 (2)	−0.007 (2)

### Geometric parameters (Å, º)

**Table:**

C1—C6	1.381 (4)	Co1—O1'	2.20 (2)
C1—C2	1.383 (3)	N2—S1	1.5941 (18)
C1—H1A	0.9300	N4—H4B	0.8600
C2—C3	1.385 (3)	N4—H4C	0.8600
C2—S1	1.760 (2)	O2—S1	1.4412 (17)
C3—C4	1.380 (4)	O3—S1	1.4470 (17)
C3—H3A	0.9300	O1—C11	1.216 (13)
C4—C5	1.390 (4)	C11—N5	1.342 (10)
C4—H4A	0.9300	C11—H11A	0.9300
C5—N4	1.370 (3)	C13—N5	1.457 (9)
C5—C6	1.396 (4)	C13—H13A	0.9602
C6—H6A	0.9300	C13—H13B	0.9597
C7—N3	1.333 (3)	C13—H13C	0.9600
C7—N1	1.360 (3)	C13—H12E	0.8765
C7—N2	1.364 (3)	C12—N5	1.489 (11)
C8—N3	1.339 (3)	C12—H12A	0.9599
C8—C9	1.378 (3)	C12—H12B	0.9600
C8—H8A	0.9300	C12—H12C	0.9601
C9—C10	1.373 (4)	O1'—C11'	1.244 (17)
C9—H9A	0.9300	C11'—N5'	1.694 (14)
C10—N1	1.327 (3)	C11'—H11B	0.9593
C10—H10A	0.9300	C13'—N5'	1.386 (13)
Co1—O1^i^	2.065 (19)	C13'—H13D	0.9600
Co1—O1	2.065 (19)	C13'—H13E	0.9600
Co1—N1	2.121 (2)	C13'—H13F	0.9600
Co1—N1^i^	2.121 (2)	C12'—N5'	1.458 (13)
Co1—N2	2.1460 (18)	C12'—H12D	0.9597
Co1—N2^i^	2.1460 (18)	C12'—H12E	0.9601
Co1—O1'^i^	2.20 (2)	C12'—H12F	0.9601
			
C6—C1—C2	120.6 (2)	N5—C13—H13B	107.4
C6—C1—H1A	119.7	H13A—C13—H13B	109.5
C2—C1—H1A	119.7	N5—C13—H13C	106.4
C1—C2—C3	118.7 (2)	H13A—C13—H13C	109.4
C1—C2—S1	120.66 (19)	H13B—C13—H13C	109.5
C3—C2—S1	120.62 (18)	N5—C13—H12E	53.8
C4—C3—C2	120.9 (2)	H13A—C13—H12E	77.9
C4—C3—H3A	119.6	H13B—C13—H12E	85.1
C2—C3—H3A	119.6	H13C—C13—H12E	159.2
C3—C4—C5	120.9 (3)	N5—C13—H12F	63.8
C3—C4—H4A	119.6	H13A—C13—H12F	83.9
C5—C4—H4A	119.6	H13B—C13—H12F	166.5
N4—C5—C4	120.8 (3)	H13C—C13—H12F	65.9
N4—C5—C6	121.2 (3)	H12E—C13—H12F	96.5
C4—C5—C6	117.9 (2)	N5—C12—H12A	157.8
C1—C6—C5	121.0 (2)	N5—C12—H12B	88.6
C1—C6—H6A	119.5	H12A—C12—H12B	109.5
C5—C6—H6A	119.5	N5—C12—H12C	74.6
N3—C7—N1	125.2 (2)	H12A—C12—H12C	109.5
N3—C7—N2	125.86 (19)	H12B—C12—H12C	109.5
N1—C7—N2	108.91 (19)	N5—C12—H13E	125.9
N3—C8—C9	123.7 (2)	H12A—C12—H13E	53.6
N3—C8—H8A	118.2	H12B—C12—H13E	70.9
C9—C8—H8A	118.2	H12C—C12—H13E	159.1
C10—C9—C8	117.0 (2)	N5—C12—H13F	153.6
C10—C9—H9A	121.5	H12B—C12—H13F	117.7
C8—C9—H9A	121.5	H12C—C12—H13F	92.7
N1—C10—C9	121.3 (2)	H13E—C12—H13F	70.1
N1—C10—H10A	119.3	C11—N5—C13	141.8 (7)
C9—C10—H10A	119.3	C11—N5—C12	99.2 (6)
O1^i^—Co1—O1	180.0 (10)	C13—N5—C12	119.0 (6)
O1^i^—Co1—N1	84.2 (3)	C11—N5—H12E	149.4
O1—Co1—N1	95.8 (3)	C12—N5—H12E	95.2
O1^i^—Co1—N1^i^	95.8 (3)	C11—N5—H12F	132.3
O1—Co1—N1^i^	84.2 (3)	C13—N5—H12F	49.4
N1—Co1—N1^i^	180.00 (11)	C12—N5—H12F	92.0
O1^i^—Co1—N2	89.0 (6)	H12E—N5—H12F	73.5
O1—Co1—N2	91.0 (6)	C11'—O1'—Co1	124.5 (17)
N1—Co1—N2	62.59 (7)	O1'—C11'—N5'	87.5 (14)
N1^i^—Co1—N2	117.41 (7)	O1'—C11'—H11B	124.6
O1^i^—Co1—N2^i^	91.0 (6)	N5'—C11'—H11B	143.7
O1—Co1—N2^i^	89.0 (6)	N5'—C13'—H12A	99.0
N1—Co1—N2^i^	117.41 (7)	N5'—C13'—H13D	115.4
N1^i^—Co1—N2^i^	62.59 (7)	H12A—C13'—H13D	126.7
N2—Co1—N2^i^	180.0	N5'—C13'—H13E	108.1
O1^i^—Co1—O1'^i^	10.6 (8)	H12A—C13'—H13E	95.5
O1—Co1—O1'^i^	169.4 (8)	H13D—C13'—H13E	109.5
N1—Co1—O1'^i^	94.1 (5)	N5'—C13'—H13F	104.7
N1^i^—Co1—O1'^i^	85.9 (5)	H13D—C13'—H13F	109.5
N2—Co1—O1'^i^	90.2 (7)	H13E—C13'—H13F	109.5
N2^i^—Co1—O1'^i^	89.8 (7)	N5'—C12'—H13A	154.8
O1^i^—Co1—O1'	169.4 (8)	N5'—C12'—H12B	60.6
O1—Co1—O1'	10.6 (8)	H13A—C12'—H12B	117.7
N1—Co1—O1'	85.9 (5)	N5'—C12'—H12C	54.9
N1^i^—Co1—O1'	94.1 (5)	H13A—C12'—H12C	150.1
N2—Co1—O1'	89.8 (7)	H12B—C12'—H12C	75.3
N2^i^—Co1—O1'	90.2 (7)	N5'—C12'—H12D	131.7
O1'^i^—Co1—O1'	180.000 (3)	H13A—C12'—H12D	66.3
C10—N1—C7	117.6 (2)	H12B—C12'—H12D	78.5
C10—N1—Co1	147.50 (17)	H12C—C12'—H12D	92.6
C7—N1—Co1	94.85 (14)	N5'—C12'—H12E	92.3
C7—N2—S1	123.96 (15)	H13A—C12'—H12E	62.9
C7—N2—Co1	93.64 (13)	H12B—C12'—H12E	85.1
S1—N2—Co1	142.24 (11)	H12C—C12'—H12E	146.9
C7—N3—C8	115.21 (19)	H12D—C12'—H12E	109.5
C5—N4—H4B	120.0	N5'—C12'—H12F	102.2
C5—N4—H4C	120.0	H13A—C12'—H12F	83.3
H4B—N4—H4C	120.0	H12B—C12'—H12F	158.7
O2—S1—O3	115.11 (11)	H12C—C12'—H12F	84.4
O2—S1—N2	113.67 (10)	H12D—C12'—H12F	109.5
O3—S1—N2	104.78 (10)	H12E—C12'—H12F	109.5
O2—S1—C2	107.31 (11)	C13'—N5'—C12'	119.6 (8)
O3—S1—C2	108.17 (11)	C13'—N5'—C11'	138.9 (8)
N2—S1—C2	107.48 (10)	C12'—N5'—C11'	101.2 (8)
C11—O1—Co1	129.0 (12)	C13'—N5'—H12B	70.6
O1—C11—N5	157.5 (12)	C12'—N5'—H12B	57.6
O1—C11—H11A	101.2	C11'—N5'—H12B	137.0
N5—C11—H11A	101.2	C13'—N5'—H12C	87.5
O1—C11—H11B	118.0	C12'—N5'—H12C	49.6
N5—C11—H11B	84.2	C11'—N5'—H12C	126.1
N5—C13—H13A	114.5	H12B—N5'—H12C	71.3

Symmetry code: (i) −*x*, −*y*+1, −*z*.

### Hydrogen-bond geometry (Å, º)

**Table:**

*D*—H···*A*	*D*—H	H···*A*	*D*···*A*	*D*—H···*A*
N4—H4*B*···O3^ii^	0.86	2.31	3.112 (3)	155
N4—H4*C*···O3^iii^	0.86	2.27	2.951 (3)	136

Symmetry codes: (ii) *x*, −*y*+3/2, *z*+1/2; (iii) −*x*+1, *y*+1/2, −*z*+1/2.
